# Codivergence of Mycoviruses with Their Hosts

**DOI:** 10.1371/journal.pone.0022252

**Published:** 2011-07-29

**Authors:** Markus Göker, Carmen Scheuner, Hans-Peter Klenk, J. Benjamin Stielow, Wulf Menzel

**Affiliations:** DSMZ – German Collection for Microorganisms and Cell Cultures, Braunschweig, Germany; University of California Merced, United States of America

## Abstract

**Background:**

The associations between pathogens and their hosts are complex and can result from any combination of evolutionary events such as codivergence, switching, and duplication of the pathogen. Mycoviruses are RNA viruses which infect fungi and for which natural vectors are so far unknown. Thus, lateral transfer might be improbable and codivergence their dominant mode of evolution. Accordingly, mycoviruses are a suitable target for statistical tests of virus-host codivergence, but inference of mycovirus phylogenies might be difficult because of low sequence similarity even within families.

**Methodology:**

We analyzed here the evolutionary dynamics of all mycovirus families by comparing virus and host phylogenies. Additionally, we assessed the sensitivity of the co-phylogenetic tests to the settings for inferring virus trees from their genome sequences and approximate, taxonomy-based host trees.

**Conclusions:**

While sequence alignment filtering modes affected branch support, the overall results of the co-phylogenetic tests were significantly influenced only by the number of viruses sampled per family. The trees of the two largest families, *Partitiviridae* and *Totiviridae*, were significantly more similar to those of their hosts than expected by chance, and most individual host-virus links had a significant positive impact on the global fit, indicating that codivergence is the dominant mode of virus diversification. However, in this regard mycoviruses did not differ from closely related viruses sampled from non-fungus hosts. The remaining virus families were either dominated by other evolutionary modes or lacked an apparent overall pattern. As this negative result might be caused by insufficient taxon sampling, the most parsimonious hypothesis still is that host-parasite evolution is basically the same in all mycovirus families. This is the first study of mycovirus-host codivergence, and the results shed light not only on how mycovirus biology affects their co-phylogenetic relationships, but also on their presumable host range itself.

## Introduction

Parasites are uniformly characterized by close ecological interactions with their hosts, but are a phylogenetically heterogeneous and diverse assemblage of multi- and unicellular biological entities. Pathogens such as viruses exhibit many parasite-like traits [1], [2] as they frequently show a high degree of host specialization and are much smaller than their hosts, thus reproducing more rapidly and in larger numbers. Mycoviruses have been ubiquitously reported from the fungal kingdom [3]–[5] and from the viral families *Barnaviridae*, *Birnaviridae*, *Chrysoviridae*, *Cystoviridae*, *Metaviridae*, *Partitiviridae*, *Pseudoviridae*, *Reoviridae* and *Totiviridae*. However, *Birnaviridae* and *Cystoviridae*, listed by [3] and [4] as infecting fungal hosts have at the time of writing not been deposited in the INSDC databases, nor are these two families listed in [6] or in the current ICTV master species list 2009 (version 9; http://talk.ictvonline.org/files/ictv_documents/m/msl/1231.aspx downloaded on March 30^th^ 2011) as viral genera infecting fungi. An important criterion for demarcating virus families is the number of segments in their genomes [6]. Lower taxa are mainly demarcated by amino acid sequence similarity, i.e. 65–100% between virus strains of the same species, 55–65% between species of the same genus, and 35–55% between genera of the same family, but other criteria are also applied (polythetic taxonomy) [6].

Viruses infecting fungi mostly consist of isometric (icosahedral) or, in the case of *Mycoreovirus* (*Reoviridae*), of spherical double-shelled particles 25–80 nm in diameter, and possess segmented double stranded RNA (dsRNA) or linear positive single stranded RNA (ss(+)RNA) genomes, but seldom an envelope [4], [6]–[8]. Less complex, simpler mycoviruses with non-encapsulated, naked dsRNA genomes, are known from *Endornaviridae* and *Narnaviridae* only [8]–[12]. Unlike encapsulated RNA, naked dsRNA located in pleomorphic vesicles is a rare exception among mycoviruses, currently known only from *Hypoviridae* [8], [13], [14]. While almost all mycoviruses replicate cytoplasmatically, the genomes of the genus *Mitovirus* (*Narnaviridae*) evince mitochondrial genetic code [11], [15]–[17]. Additionally, a dsDNA virus, the unclassified genus *Rhizidiovirus*, is occasionally reported as being isolated from a fungus, but has never been sequenced.

While the majority of mycovirus-containing families do not exclusively infect fungi, but a wide range of hosts such as prokaryotes, plants and *Metazoa* [18]–[22], three families exclusively infect fungi; these are *Barnaviridae*, *Hypoviridae*, and *Pseudoviridae*. *Narnaviridae* contain recently added records from plants (e.g. ‘Grapevine associated narnavirus-1’; Genbank accession GU108586), which challenge the previous view of this family as harboring only micoviruses. Alternatively, endophytic fungi associated with plant vessels might be the real hosts of these pathogens. Using pyrosequencing, [23] detected a variety of mycoviruses in fungal strains isolated from stems of grapevine, but not as many as directly in the host plants.

Infections with fungal viruses often remain persistently undetected in their hosts, as mycoviruses are usually not associated with obvious disease symptoms [4]. Accordingly, viruses causing altered phenotypes, such as reduced growth, pigmentation, sporulation or increased virulence are therefore of particular scientific interest. Mycoviruses causing fungal hypovirulence (attenuation of fungal virulence) or debilitation as a result of an altered physiology have been studied intensively in plant-pathogenic fungi [15], [16], [24]–[26]. Changes of colony and lesion morphology in economically important, destructive pests such as *Botrytis cinerea*, *Cryphonectria parasitica*, *Ophiostoma ulmi* or *Sclerotinia scleroderma* provide convincing evidence that mycoviruses can both in- and decrease fungal pathogenicity [4], [14], [24], [27]. Unlike deleterious infections which decrease host fitness, mycoviruses may have evolved in concert with their hosts, yielding mutual benefits [3], [28]. For instance, ‘killer phenotypes’ of yeasts (e.g., *Saccharomyces cerevisiae*, *Ustilago maydis*) contain a helper-virus dependent satellite dsRNA which encodes both a toxin and immunity to this toxin, which allows them to outcompete other strains of the same species [3], [29], [30]. However, in most cases reduced host fitness caused by mycovirus infections and, hence, negative implications for a fungal host population are likely to not favor persistence of either horizontally or vertically transmitted infections [5], [27], [31], [32].

In contrast to most plant pathogenic and many animal pathogenic viruses (see, e.g., http://www.ictvdb.org/Ictv/ICD-10.htm for viruses pathogenic to humans), natural vectors transmitting mycoviruses are unknown [3], [5]. Only intracellular transmissions by hyphal anastomosis and heterokaryosis (horizontal transmission) and spread via sexually or asexually derived spores (vertical transmission) have been observed [5], [33]. Virus dissemination in mycelial networks via dolipores and septa is believed to be a passive phenomenon, as organelles easily migrate in between adjacent cells. So far, suppression of viral infections and, therefore, of transmission to the progeny is only known from *Aspergillus* section *Flavi* via asexually produced spores [28]. Also, heterokaryon incompatibility reactions preventing hyphal fusion effectively inhibit virus transmission.

One of the basic and important questions in evolutionary biology is the degree to which the diversification of parasites is linked to the diversification of their hosts [34]–[36]. Under the assumptions that viruses are host-specific and that they are transmitted only vertically (e.g., because there are no natural vectors), the phylogeny of viruses should be topologically congruent with that of their hosts, i.e. correspond to Fahrenholz' rule of strict codivergence [37]. Alternatively, combinations of events such as host switching, duplication and parasite extinction can lead to topological incongruence between the phylogenies of viruses and their hosts [38]–[40]. Here, “switching” refers to the lateral transfer of the parasite and a successful colonization of a novel host which is phylogenetically only distantly related to the previous host; if such an event was accompanied by an according parasite speciation, a “complete switch” occurred, an “incomplete switch” otherwise. “Duplication” refers to adaptive radiation of the parasite on the same host species, yielding a set of parasite sister groups with an identical host range.

Virus interspecies transmission might either require the adaptation to a new host species during the early stages of infection or largely be a random process, involving the genetic founder effect [41], [42]. An as yet non-colonized host might represent an ‘ecological license’, i.e. a previously not utilized unit of the environment that is suitable for becoming an ecological dimension of a pathogen's niche [34]. ‘Resource tracking’ describes a pattern in which a parasite is associated with a set of hosts that share a certain resource; to the extent that these hosts can be phylogenetically unrelated, host and parasite phylogenies can disagree [43]. Timm [44] contrasted Fahrenholz' rule [37] with resource tracking and hypothesized that a low probability of lateral parasite transfer to new hosts, and, hence, a low degree of resource tracking, is the main cause for topological congruence between host and parasite trees. A typical host-parasite system with little likelihood for lateral transfer is the association between pocket gophers and their chewing lice. Pocket gophers are distributed allopatrically and infrequently leave their burrows [44], hence the chewing lice have little chance to switch to new hosts. This system was frequently used to assess algorithms for statistical co-phylogenetic tests, all of which indicated a significant degree of congruence between gopher and louse phylogenies [39], [45], [46].

In this study, we assess the hypothesis that mycoviruses codiverge with their hosts using state-of-the-art statistical tests [47]–[49]. We investigate the evolutionary dynamics of all viral families containing mycoviruses by inferring virus phylogenies from their genome sequences and comparing them to the phylogenies of their hosts. The latter are approximated using the taxonomic classification of the hosts [48], [50], [51], but in contrast to these earlier studies we assess the effect of distinct approaches to inferring branch lengths from the classifications. Because of the comparatively low degree of sequence similarity even within virus families, sequence alignment and subsequent phylogenetic inference might be difficult [52]–[55]. Accordingly, we also determine the sensitivity of the co-phylogenetic tests to the settings used for filtering the gene alignments as collected from the viral genomes. To the best of our knowledge, this is the first co-phylogenetic study of mycoviruses and their hosts.

## Methods

### Data collection and assembly of supermatrices

Sequence data for all virus families that include at least five mycoviruses with distinct ‘ORGANISM’ entries were downloaded from Genbank on February 14^th^ 2011. Sets of sequences representative of the genome of each virus taxon were created by storing all accessions containing the same ‘ORGANISM’ entry in a separate file. Accordingly, the protein sequences from originally 15 *Chrysoviridae* genomes, 12 *Endornaviridae* genomes, 7 *Hypoviridae* genomes, 25 *Narnaviridae* genomes, 59 *Partitiviridae* genomes and 57 *Totiviridae* genomes could be used for assembling supermatrices (but some were removed later on in one of the filtering steps).

The phylogenomic pipeline used for assembling supermatrices (i.e., the concatenation of potentially many genes) is the one applied in [56] and [57] with a single modification for removing genomes with poor sequence overlap. For each viral family a genome-against-genome protein BLAST search was performed using BLAST version 2.2.17 [58] with soft masking instead of complexity filtering. To determine orthologs, BLAST e-values were transformed using a re-implementation of the OrthoMCL algorithm [59] in conjunction with MCL version 08-312 (http://micans.org/mcl/) using an inflation parameter of 2.0. OrthoMCL clusters containing inparalogs were reduced by selecting the most ‘central’ of several sequences from the same genome, that is, the sequence with the highest sum of within-cluster BLAST scores. The reduced OrthoMCL clusters were aligned using MUSCLE version 3.7 [60].

The program scan_orphanerrs from the RASCAL package version 1.3.4 [54] was applied to detect orphan (i.e., overall poorly aligned) sequences within the alignments. After removal of orphan sequences (if present), poorly aligned columns and divergent regions were eliminated with GBLOCKS version 0.91b [52] using a minimum block length of two amino acids and allowing gap positions in all sequences. Prior to concatenating the single-gene alignments, the OrthoMCL clusters were checked for pairs of taxa without co-occurrence of genes in any cluster. Taxa were sorted in decreasing order of their total number of such pairs and removed iteratively until all remaining pairs of taxa included sequences that co-occurred in at least one cluster. In the case of ties, preferably those taxa whose sequences, on average, occurred in the more sequence-rich (better sampled) clusters were kept. Filtered OrthoMCL cluster alignments containing at least four sequences were concatenated to form a supermatrix for phylogenetic analysis. The dependency of the (co-)phylogenetic results on these filtering steps was assessed by omitting either RASCAL or GBLOCKS filtering or both and conducting phylogenetic inference also for the resulting alternative matrices.

### Phylogenetic inference

The Pthreads-parallelized RAxML version 7.2.8 software [61] was used for inferring trees from the supermatrices under the maximum-likelihood criterion [62]. The best substitution model for each supermatrix was determined by comparing the resulting log likelihoods for all models implemented in RAxML version 7.2.8 (for performance reasons, except GTR) applied to a parsimony starting tree. Under the respective optimal model, 100 rounds of rapid bootstrapping [63] with subsequent search for the best tree were conducted for each supermatrix.

As a proxy for host phylogenies, we used the current (February 28^th^ 2011) release of the NCBI classification for calculating taxonomy-based distances, an approach introduced by [50] and also applied in [48] and [51], which used classification-based distances also for the parasites. Patristic (path-length) distances between the hosts were inferred using the method applied in these three publications. In the context of taxonomic classifications, the patristic distance *d_P_(X,Y)* between two taxa *X* and *Y* is equivalent to the number of taxa (including itself) to which *X* belongs but not *Y* plus the number of taxa (including itself) to which *Y* belongs but not *X*. Let *t(A)* denote a function that returns the set of parent taxa of taxon *A* (including itself), *d_P_(X,Y)* is defined as follows:
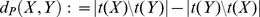
(1)


Such ‘patristic’ distances *d_P_* are additive [64] because they are derived from a tree [50] but seldom ultrametric [65], even if *X* and *Y* have the same taxonomic rank, because the number of taxonomic ranks in use differs between distinct lineages (see supplementary File S1).

Three potential sources of biases must considered when using classification-based distances in co-phylogenetic analyses: (i) the classification might not reflect the natural relationships because it contains non-monophyletic groups; (ii) the distances may contain many ties because classification trees can be rather unresolved due to the limited number of taxonomic ranks; and (iii) the distances can only roughly be interpreted in biological terms (e.g., they do not represent the amount of character change). Dubious taxonomic classifications (i) are of general importance but unlikely to affect precisely those host taxa studied here; this issue is discussed below. While the problem (ii) is also unlikely to have a significant impact on the current study because most included host taxa are very distantly related, we address issue (iii) explicitly by inferring classification-based distances using three additional formulas and assessing the sensitivity of outcome of the co-phylogenetic tests to the distance formula used. ‘Quasi-patristic’ distances attempt to scale patristic distances according to the number of taxonomic ranks in use for each considered pair of taxa:
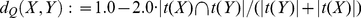
(2)


Distances derived from this formula are usually not additive, but are expected to deviate less from ultrametricity than *d_p_* because the scaling is applied. For the datasets examined in the current study, these assertions are confirmed in File S1, using quartet statistics applied in [66] to assess (deviation from) additivity and additional triplet statistics to assess (deviation from) ultrametricity.

‘Theory’ distances apply the formula derived by Lin [67] from information theory for semantic similarities in taxonomies:
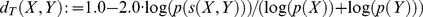
(3)where *s(X,Y)* is the smallest parent taxon of both *X* and *Y* and *p(A)* is the probability of taxon *A* as derived from its relative frequency, i.e. the number of leaves in *A* divided by the total number of leaves in the classification tree. If these numbers refer to a classification which has been reduced to the taxa of interest and their parent taxa, the ‘theory’ formula *d_T_* yields ultrametric distances (see S1). The fourth distance formula applied here, ‘first mismatch’, refers to the number of parent taxa (potentially including the taxon itself) in common between each pair of taxa *X* and *Y* (*X*≠*Y*) of interest:
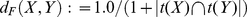
(4)


Here, *d_F_(A,A)* needs to be defined separately as 0.0 for all *A* to obtain proper distances. These *d_F_* distances are also ultrametric (see S1). Compared to (1) and (2), a drawback of formula (4) is that the distance between a taxon and each of its parent taxa is zero, but this is not of practical relevance to the current study (nor to any other study in which only distances between taxa of the same rank are inferred).

Deriving the four types of distances from the NCBI classification is implemented in an unpublished script available from the corresponding author upon request. With several distance formulas available, sensitivity of the co-phylogenetic outcome to distinct biological interpretations of the host classifications can be investigated.

### Co-phylogenetic tests and assessment of parameter sensitivity

Each combination of maximum-likelihood parasite tree and classification-based host distance matrix was subjected to the ParaFit co-phylogenetic test [46] as implemented in AxParafit [48]. Customized scripts functionally equivalent to CopyCat [50], e.g., applying AxPcoords [48] for converting distance matrices to eigenvectors (principal coordinates), were used for batch processing the data. Patristic distances were inferred from the virus phylogenies using the newick.tcl script (http://www.goeker.org/mg/distance/). ParaFit uses pair-wise or patristic distances to test the global null hypothesis (‘GH0’ in the following) that the agreement between the trees is not higher than expected by chance, given the actual associations (links) between hosts and parasites [46]. In contrast to other co-phylogenetic tests, ParaFit further estimates the contribution of each individual host-parasite link to the global fit between the matrices to test the individual null hypothesis (‘IH0’) that any given contribution is not different from random (i.e., the link could as well be omitted). We will term links for which IH0 was accepted ‘non-significant’, ‘significant’ otherwise. Significance testing is based on permuting the rows of the association matrix, not the trees. In contrast to other co-phylogenetic tests, type I and type II error ratios of ParaFit have been explored in extensive simulation studies [46].

The effect of modifying the pipeline's settings on the results from phylogenetic inference was assessed using a multiple linear regression as implemented in R version 2.12.1 [68] with the average bootstrap support (BS) as dependent and the original supermatrix dimensions (number of viruses and number of ortholog clusters) as well as alignment filtering settings as independent variables. The parameter sensitivity of the co-phylogenetic analyses was tested using the host distance formula and either the above-mentioned independent variables or the average BS as explanatory variables and the proportion of significant links as the response variable. R provides a step-wise regression procedure to eliminate insignificant variables based on the Akaike information criterion (AIC; see pp. 128–129 in [69]). The AIC aims achieving a balance between model likelihood and model simplicity (the number of parameters used to explain the data), in accordance with the principle known as ‘Ockham's razor’ (see pp. 499–525 in [70], or [71]). In each step, a variable which (according to the AIC) does not significantly improve the fit of the regression model to the data is removed and all regression parameters are recomputed. The step-wise elimination stops when all remaining explanatory variables make a significant contribution. We used ‘both’ as stepwise search mode and considered all possible interactions between the explanatory variables. Afterwards, stepwise manual removal of insignificant variables (p>0.01) was applied, as described, e.g. on p. 442 in [72]. R automatically recodes qualitative variables into a set of binary variables (see pp. 46–47 in [70]) suitable for linear regression. All variables representing counts (e.g., number of viruses) were log-transformed, whereas all proportions (e.g., average BS) were arcsin-transformed, as recommend in p. 386 in [73]. The same version of R [68] was used to visualize the distribution of genes over the viral genomes and clusters of orthologs as heatmaps (see supplementary File S3).

In addition to ParaFit, other methods for testing codivergence hypotheses are available [47]; for details on our selection of methods see [49], which used exactly the same co-phylogenetic tools for the same tasks. They were here applied to selected host-parasite datasets only, depending on the results of the ParaFit tests and the parameter sensitivity analysis.

TreeFitter [45] uses generalized parsimony to explore different cost combinations for each of the four types of events that might occur in the natural history of associated groups of organisms [45], [74]: codivergence, host switching, duplication or intra-host divergence of the parasite, and sorting or extinction of the parasite lineage. Given a predefined combination of costs for each of these events, TreeFitter [45], [75] attempts to minimize the global cost; permutation tests can be applied to determine the number of times an equally low or lower total cost is found for randomized associations and thus the probability (p value) of the null hypothesis that the fit is not better than expected by chance. To determine the event cost combination that best explains the data we followed the procedure outlined in [75], who presented the results of this permutation-based approach for six hypothetical evolutionary patterns. The overall best combinations of event costs are held to be those that yield the lowest probability of the null hypothesis. Codivergence and sorting events were assigned zero and unit costs (1.0), respectively, whereas switching and duplication costs were varied between 0.0 and 10.0 in increments of 0.5 [49], [75]. For each combination of costs, 10,000 permutations of the original associations were conducted [49], [75]. Where polytomies were present in the host trees (which were derived from the NCBI classification), these were resolved randomly to enable input into TreeFitter. This was needed for *Chrysoviridae*, *Partitiviridae* and *Totiviridae*. Because TreeFitter v1.1 does not allow multiple hosts per parasite, only the first host of multiple-host parasites was kept. However, this reduction usually only affected host species from the same genus (*Aspergillus*, *Heterobasidion*) and is thus unlikely to affect the test results.

TreeMap 2.02beta (http://www.it.usyd.edu.au/~mcharles/) also implements tree reconciliation of host and associate trees [39] and particularly the Jungles algorithm [76]. However, running time may be prohibitive even for moderately-sized datasets [49], and TreeFitter might be better justified in theoretical terms than the algorithm implemented in TreeMap [75]. We thus used TreeMap to visualize host-parasite tanglegrams only. In contrast to ParaFit, TreeFitter and TreeMap need rooted input trees. Outgroup taxa were deliberately not included in the current study because (due to the low degree of sequence similarity between distinct virus families; see above) they would decrease the overlap between the viral genomes regarding the clusters of orthologs. Hence, in order to apply a neutral, host-independent rooting, the midpoint rooting method [77], [78] as implemented in PAUP* version 4.0b10 [79] was used to root all parasite trees. Reduction of multiple hosts and random resolving of polytomies for TreeMap was conducted as for TreeFitter.

## Results

### Overall (co-)phylogenetic results and their parameter sensitivity

The characteristics of the obtained supermatrices and the resulting trees are shown in supplementary file S2. Twelve viruses were removed before ortholog determination because none of their accessions contained protein sequences (see supplementary file S2). Re-annotating Genbank entries was beyond the scope of the present study, and most of these accessions comprised rather incomplete genome fragments, sometimes only from 5′ and 3′ untranslated regions. Another 15 viruses had to be deleted because their genes were only present in clusters of orthologs that comprised less than four distinct viruses. Note that the minimal size of a nontrivial unrooted tree is four leaves; such small clusters of genes thus would add little information in phylogenetic inference. Finally, five viruses were removed by the algorithm for removing genomes with poor overall sequence co-occurrence (‘*Debaryomyces hansenii* virus JB-2008’ and ‘Grapevine associated totivirus-3’ from the *Totiviridae* dataset, ‘Grapevine associated chrysovirus-2’, ‘Grapevine associated chrysovirus-3’ and ‘Grapevine associated chrysovirus-4’ from the *Chrysoviridae* dataset). These virus genomes did not share even a single cluster of orthologs with the majority of the genomes in the respective dataset. Supplementary file S3 visualizes the presence or absence of genes in each viral genome and cluster of orthologs as heatmaps and demonstrates the reasons for the deletion of each excluded virus taxon. It also shows that the deleted viruses did not form sufficiently large groups themselves, which could have been subjected to separate phylogenetic analyses.

The number of clusters of orthologs that could be obtained for each virus family is as expected; for instance, for the non-encapsulated *Narnaviridae* no coat proteins were found (i.e., the resulting supermatrix was not that ‘super’ at all). In addition to the RNA-dependent RNA polymerase, which is, for obvious reasons, present in all examined virus families, only coat proteins were present with the exception of a ‘putative protease’ in the case of *Chrysoviridae*. The number of clusters of orthologs available for phylogenetic inference ranged from one to six (*Partitiviridae*), the number of characters from five to 6237, and the proportion of gaps or missing character states from 2% to 85%. The number of characters was, of course, also dependent on the filtering of the alignments, with most characters present in unfiltered alignments, followed by RASCAL-only filtering, RASCAL+GBLOCKS filtering, and filtering with GBLOCKS alone. The best ML models selected varied between the supermatrices and were not virus-specific with the single exception of RTREVF (*Partitiviridae* filtered with both RASCAL and GBLOCKS).

The results of the test with AxParafit are shown in supplementary file S2. The GH0 was accepted for *Chrysoviridae* and *Hypoviridae* under all settings and for *Endoviridae* and *Narnaviridae* under most. The individual links were all insignificant for the former two families and almost always insignificant for the *Endornaviridae* and *Narnaviridae*. In contrast, GH0 was rejected for *Partitiviridae* and *Totiviridae* under all conditions. The proportion of significant links ranged between 27% and 70% (median, 52%) for *Partitiviridae* and between 38% and 91% (median, 74%) for *Totiviridae*.

The result from a multiple linear regression with the average BS as dependent variable is shown in Table 1. The original number of viruses (before alignment filtering) as well as filtering with GBLOCKS have a significant negative effect, whereas filtering with RASCAL has a strong positive effect, followed by filtering with both RASCAL and GBLOCKS. Other explanatory variables were eliminated as insignificant. If the total number of characters and the average number of determined characters per taxon were taken as dependent variables, only GBLOCKS filtering had a significant and negative effect (data not shown). The result from a multiple linear regression with the proportion of significant links as dependent variable is shown in Table 2. Only the original total number of viruses had a significant (and positive) impact on the outcome of the ParaFit test.

**Table 1:** Result from a linear regression after step-wise variable elimination according to the AIC followed by step-wise manual removal of insignificant variables (p>0.01) with the arcsin-transformed average bootstrap support of the maximum-likelihood virus trees as dependent variable and the original dataset sizes and the alignment filtering settings as explanatory variables.

Coefficient	Estimate	Standard error	t value	Probability (>|t|)
Intercept	1.720	0.123	13.992	8.61e-12***
Original number of virus taxa, logarithmized	−0.212	0.038	−5.618	1.69e-05***
RASCAL filtering on	0.271	0.064	4.252	0.00039***
GBLOCKS filtering on	−0.186	0.064	−2.924	0.00840**

The significance codes are: ‘***’, 0.001; ‘**’, 0.01. The residual standard error was 0.1559 on 20 degrees of freedom. The multiple R^2^ was 0.7442, the adjusted R^2^ 0.7058.

**Table 2:** Result from a linear regression after step-wise variable elimination according to the AIC followed by step-wise manual removal of insignificant variables (p>0.01) with the arcsin-transformed proportion of significant links according to each ParaFit test as dependent variable and the original dataset sizes, alignment filtering settings and host distance formulas as explanatory variables.

Coefficient	Estimate	Standard error	t value	Probability (>|t|)
Intercept	−0.647	0.113	−5.739	1.16e-07***
Original number of virus taxa, logarithmized	0.367	0.037	9.897	3.03e-16***

The significance code is: ‘***’, 0.001. The residual standard error was 0. 3067 on 94 degrees of freedom. The multiple R^2^ was 0.5103, the adjusted R^2^ 0.5051.

### The co-phylogenetic relationships in detail

**Figure 1:**
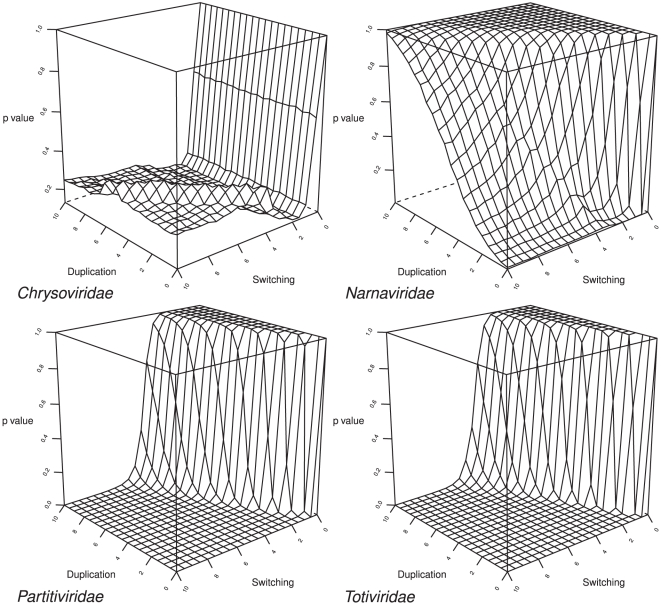
Results of the cost-space exploration with TreeFitter for *Chrysoviridae* (upper left), *Narnaviridae* (upper right), *Partitiviridae* (lower left) and *Totiviridae* (lower right). For each family, the resulting p values are plotted over the respective combination of duplication (parasite speciation on a single host) and switching (lateral transfer of the parasite) cost. Duplication and switching costs were varied between 0.0 and 10.0 in increments of 0.5. Those p values at most as large as the chosen threshold (α = 0.05) indicate a set of evolutionary event costs which explains the data significantly better than random.

The following in-depth studies of each virus family using TreeFitter and TreeMap in addition to AxParafit focus on the datasets that underwent both RASCAL and GBLOCKS filtering; significance of links is denoted in the tanglegrams according to the ‘theory’ host distances. We do not depict TreeFitter results and tanglegrams for *Endornaviridae* (comprising the single genus *Endornavirus*) and *Hypoviridae* (comprising the single genus *Hypovirus*) because they were trivial due to the small size of the dataset resulting under these settings. For the remaining four families, plots of the resulting p values over the duplications and switching costs are depicted in Fig. 1.

**Figure 2:**
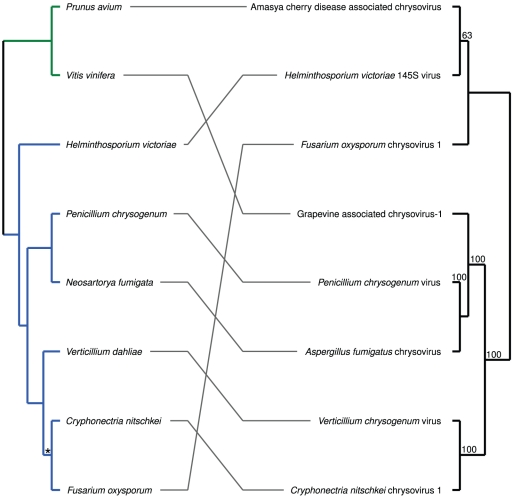
Tanglegram for the *Chrysoviridae* and their hosts. The parasite supermatrix was constructed using RASCAL and GBLOCKS alignment filtering, and the ‘theory’ host distances were used. All links were insignificant according to the ParaFit test, which also accepted the global null hypothesis of no correspondence between host and *Chrysoviridae* phylogenies. The numbers on the branches within the parasite tree are maximum-likelihood bootstrap values ≥60%. Host branches are colored according to their deep taxonomic affiliations: blue, *Fungi*; light blue, *Ascomycota*. Stars on the host branches indicate those that were obtained by randomly resolving polytomies; all other branches were derived from the host classification.

**Figure 3:**
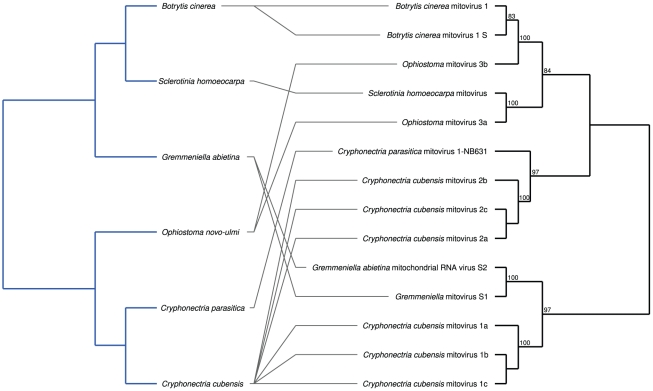
Tanglegram for the *Narnaviridae* and their hosts. The parasite supermatrix was constructed using RASCAL and GBLOCKS alignment filtering, and the ‘theory’ host distances were used. All links were insignificant according to the ParaFit test, which also accepted the global null hypothesis of no correspondence between host and *Narnaviridae* phylogenies. The numbers on the branches within the parasite tree are maximum-likelihood bootstrap values ≥60%. Host branches are coloured according to their deep taxonomic affiliations: blue, *Fungi* (light blue, *Ascomycota*; dark blue, *Basidiomycota*); green, *Viridiplantae*.

For *Chrysoviridae*, comprising the single genus *Chrysovirus*, TreeFitter analysis did not yield any significant results (α = 0.05) irrespective of the cost settings (Fig. 1). The tanglegram in Fig. 2 depicts the all-insignificant links between the *Chrysoviridae* and their hosts. In TreeFitter cost-space exploration, *Narnaviridae* obtained significant results (α = 0.05) for all positive switching costs combined with low duplication costs (Fig. 1). The tanglegram in Fig. 3 shows the all-insignificant links between the *Narnaviridae* and their hosts. (*Narnaviridae* comprises the two genera *Mitovirus* and *Narnavirus*, but the latter was removed during alignment filtering under these settings.)

**Figure 4:**
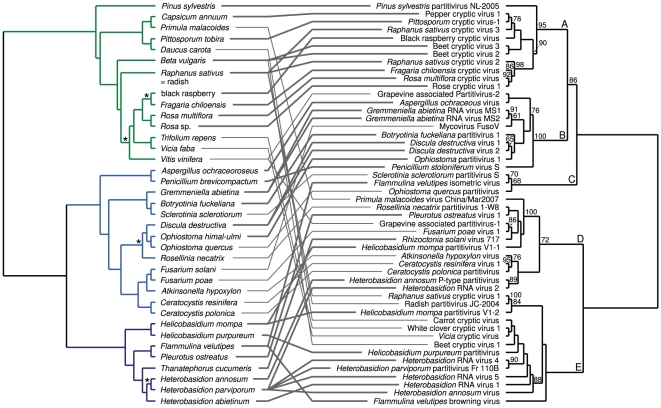
Tanglegram for the *Partitiviridae* and their hosts. The parasite supermatrix was constructed using RASCAL and GBLOCKS alignment filtering, and the ‘theory’ host distances were used. Most (69%) links were significant according to the ParaFit test, which also rejected the global null hypothesis of no correspondence between host and *Partitiviridae* phylogenies. The numbers on the branches within the parasite tree are maximum-likelihood bootstrap values ≥60%. ‘A’, ‘B’, ‘C’, ‘D’ and ‘E’ denote the major virus clades as discussed in the text. Host branches are colored according to their deep taxonomic affiliations: blue, *Fungi* (light blue, *Ascomycota*; dark blue, *Basidiomycota*); green, *Viridiplantae*. Stars on the host branches indicate those that were obtained by randomly resolving polytomies; all other branches were derived from the host classification.

For *Partitiviridae*, TreeFitter analysis yielded significant results (α = 0.05) for all duplications costs if combined with positive switching costs (which needed to be somewhat higher for higher duplication costs) (Fig. 1). Fig. 4 shows the tanglegram for the *Partitiviridae* and their hosts. Besides the genus *Partitivirus*, *Partitiviridae* comprises *Cryspovirus* and *Alphacryptovirus* (no sequences are available for *Betacryptovirus*); sequences of the former were removed during alignment filtering, whereas the latter is not shown to be monophyletic (e.g., the three ‘Beet cryptic virus’ exemplars do not group together). The parasite phylogeny contained a clade supported by 95% BS (clade ‘A’) that was exclusively associated with parasites of green plants (*Viridiplantae*); all of these links were significant. The topology within clade ‘A’ largely follows the host phylogeny; for instance, the single gymnosperm (*Pinus*) virus is sister of all angiosperm viruses, and three of the four *Rosaceae* parasites group together as well as the two beet (*Beta vulgaris*) viruses. The sister group of the *Viridiplantae* viruses, clade ‘B’, achieved 100% BS and comprised exclusively *Ascomycota* (*Pezizomycotina*) parasites with significant links except for two viruses insignificantly associated with *Fusarium* (also *Ascomycota*) and *Vitis* (*Viridiplantae*), respectively. The remaining three annotated clades, ‘C’, ‘D’ and ‘E’, are not that well supported (68%, 72%, and <50% BS, respectively) and display an inverse pattern of host relationships, i.e. exclusively contain viruses with significant links to *Basidiomycota* interspersed with parasites with insignificant associations to *Ascomycota* or *Viridiplantae*.

**Figure 5:**
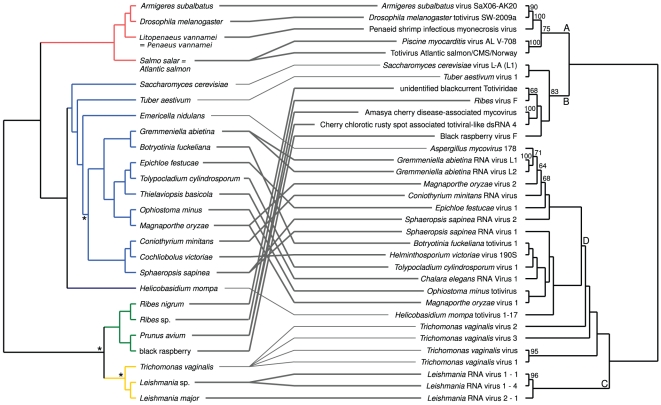
Tanglegram for the *Totiviridae* and their hosts. The parasite supermatrix was constructed using RASCAL and GBLOCKS alignment filtering, and the ‘theory’ host distances were used. Most (82%) links were significant according to the ParaFit test, which also rejected the global null hypothesis of no correspondence between host and *Totiviridae* phylogenies. The numbers on the branches within the parasite tree are maximum-likelihood bootstrap values ≥60%. ‘A’, ‘B’, ‘C’ and ‘D’ denote the major virus clades as discussed in the text. Host branches are colored according to their deep taxonomic affiliations: blue, *Fungi* (light blue, *Ascomycota*; dark blue, *Basidiomycota*); green, *Viridiplantae*; red, *Metazoa*; yellow, others. Stars on the host branches indicate those that were obtained by randomly resolving polytomies; all other branches were derived from the host classification.

The resulting pattern in TreeFitter cost-space exploration of *Totiviridae* was similar to the one of *Partitiviridae* (Fig. 1). Fig. 5 shows the tanglegram for the *Totiviridae* and their hosts. A clade (clade ‘A’) supported by 75% BS exclusively contained parasites of *Metazoa* (not assigned to a viral genus) with significant links. The topology within the clade mirrored the split of the hosts in *Arthropoda* and *Chordata*. Also, among the three arthropod parasites, the two insect viruses appeared as sister groups. Sister group of the *Metazoa* viruses was a clade supported by 83% BS (clade ‘B’) comprising four parasites (genus *Totivirus* or unassigned) with significant links to *Viridiplantae* and two with insignificant links to *Ascomycota*. A further weakly supported clade (‘C’ in Fig. 5) contained three parasites (genus *Leishmaniavirus*) of *Euglenozoa* (*Leishmania* spp.; significant links). A grade of four viruses (genus *Trichomonasvirus*) with insignificant associations to *Parabasalia* (exclusively *Trichomonas vaginalis*) led to a final clade, ‘D’, containing only *Ascomycota* viruses (genera *Totivirus* and *Victorivirus*) with significant links, the sole exception being an insignificant association with *Helicobasidium mompa* (*Basidiomycota*).

Details for all conducted tests, including information about the test results for all individual associations, are provided in the supplementary file S2.

## Discussion

### Parameter sensitivity in the detection of codivergence

Overall, little sensitivity of the co-phylogenetic tests with ParaFit to the variation of the settings used in phylogenetic inference was observed. *Partitiviridae* and *Totiviridae* were uniformly detected as co-diverging globally, as well as locally for a large proportion of hosts and viruses, whereas *Chrysoviridae* and *Hypoviridae* were uniformly considered as not having codiverged with their hosts at all. In contrast, a significant global agreement between host and parasite phylogenies, as well as a certain amount of significant individual links, was observed under some settings in the case of *Endornaviridae* and *Narnaviridae*, but not under others. However, even in the case of these two families, the number of significant links, if any, was small. Real co-divergence is likely to be low and at the margin of being detectable in these two datasets, and the ParaFit test uniformly indicated a low proportion of significant links. That 50% significant links were observed for the *Endornaviridae* after alignment filtering with RASCAL is not an exception to this rule because the test of individual links by ParaFit has an acceptable error ratio only if the global null hypothesis is rejected [46], which was not the case for these datasets. In addition to the stability of the ParaFit test results, TreeFitter and ParaFit agreed regarding the acceptance or rejection of the hypothesis of an overall agreement between host and virus phylogenies for the tested datasets (Fig. 1).

Despite the overall stability, details of the outcomes of the co-phylogenetic tests varied, and one wonders whether these dependencies can be interpreted technologically and biologically. The lack of a significant agreement between host and parasite phylogenies might either be caused by artifacts of phylogenetic inference or might simply be due to a real lack of codivergence. However, because a significant agreement between host and parasite phylogenies can not arise by magic, the fact that some method settings increase the number of significant links indicates that the resulting parasite trees became more accurate by modifying the methods in that manner. In that respect, the datasets examined here could, in principle, be used to some degree for an assessment of the accuracy of phylogenomic methods, particularly regarding alignment filtering.

In our view, the observed effects of filtering before supermatrix construction on the average BS (Table 1) are easy to explain. The number of leaves in the trees has a negative effect because, given an upper limit of the number of characters that can be sampled, the information content of the matrix decreases relative to the number of taxa to be positioned in the tree. The negative effect of GBLOCKS filtering can be interpreted in the same manner because GBLOCKS removes character information from the matrix [55]. Our RASCAL approach, in contrast, deletes complete single sequences if they are poorly aligned, thus potentially removing ‘rogue taxa’ [80], [81] whose position in the trees greatly varies in the bootstrap replicates, thus decreasing overall support. The presence of poorly aligned ‘rogue taxa’ is likely in taxa such as viruses which are characterized by comparatively low sequence similarity even within families [6]. Moreover, applying RASCAL before GBLOCKS causes the latter to remove fewer columns from the matrix (supplementary file S2), most likely because these columns otherwise appeared poorly aligned simply because of the presence of one to few poorly aligned sequences. Thus, more characters remain in the matrix, providing information for the placement of the well aligned sequences. Finally, omitting filtering entirely also results in comparatively higher bootstrap support values simply because more characters remain [53]. However, it has been observed that leaving potentially wrongly aligned characters in protein alignments can result in increased support for wrong groupings [55]; higher average support does not indicate higher accuracy. In the current study, the filtering settings did not have a significant impact on the proportion of significant links detected (Table 2). Hence, it is unlikely that alignment cleaning had, on average, either a beneficial or adversary effect on phylogenetic accuracy regarding the here examined datasets.

The importance of a sufficient amount of character information available for phylogenetic inference has been discussed particularly in the context of phylogenomics because the steady and rapid improvements in genome sequencing technology promise that genome-scale data are soon available for many organisms [82], providing the large number of characters needed to solve difficult phylogenetic problems [83]. In the case of viruses their principally small genomes of course severely limit the chances for increased character sampling, particularly in families such as *Narnaviridae* which do not even encode coat proteins. Sampling more taxa is thus the only way of improving phylogenetic accuracy [84], [85] in such cases. However, in the current study average BS values decreased with increasing numbers of viruses (Table 1, supplementary file S2), but this did not apparently affect the outcome of the co-phylogenetic tests (Table 2, supplementary file S2).

In fact, increased taxon sampling is likely to have a direct, beneficial impact on the co-phylogenetic tests. Simulations conducted by Legendre et al. [42] have shown that, given fixed proportions of codiverging and non-codiverging host-parasite pairs, the power of the global and individual ParaFit tests increases with increasing host and parasite sample sizes. This is in accordance with our observation that, as the only significant explanatory variable, the original number of viruses in each dataset has a strong positive effect on the proportion of significant links detected (Table 2). Because of this apparent effect of dataset size on the outcomes of the co-phylogenetic tests, we caution against an over-interpretation of the differences between the test results obtained for *Partitiviridae* and *Totiviridae* on the one hand and the remaining families (*Chrysoviridae*, *Endornaviridae*, *Hypoviridae*, *Narnaviridae*) on the other hand. The latter might simply be too sparsely sampled to enable the unambiguous detection of co-divergence with their hosts. At the very least, the hypothesis that there are no principal differences between all families containing mycoviruses regarding their mode of evolution relative to the evolution of their hosts is currently the most parsimonious one.

A final effect to be discussed is the formula used for deriving distances from the host classification. In contrast to earlier studies that used classifications in co-phylogenetic studies [48], [50], [51], we here varied the calculation of the branch lengths for assessing their impact on the outcome of the ParaFit test. The factor was not significant in regression analysis, indicating that modifying the formula for inferring branch lengths does not affect the overall outcome of the co-phylogenetic tests. While other formulas for classification-based distances might also be biologically reasonable, we opine that the use of the four approaches already enabled us to assess the sensitivity of co-phylogenetic tests to distinct interpretations of biological classifications regarding branch-length information. Moreover, the taxonomic classification of a certain group of organisms might only insufficiently reflect their natural relationships, for instance because it is outdated and does not incorporate results of state-of-the-art phylogenetic methods and datasets. The *Opisthokonta* hypothesis, i.e. sister-group relationship of fungi and *Metazoa* relative to other groups of multicellular organisms, relevant for the *Totiviridae* dataset (Fig. 5) , has been confirmed by the majority of multi-locus molecular phylogenetic studies [86], [87]. Also, the current higher-level classification of fungi is based on comparatively recent (multi-gene) molecular phylogenetic reconstructions and a selection of state-of-the-art phenotypic data such as ultrastructural features [88]. However, using taxonomic classifications in co-phylogenetic studies might not be advisable for other groups of organisms.

### Host-parasite codivergence in families comprising mycoviruses

Ronquist [75] created artificial datasets as exemplars for distinct combinations of events dominant in host-parasite evolution and depicted the outcomes of cost-space exploration with TreeFitter for these datasets. Via comparison with these exemplars the dominant evolutionary modes in empirical data can be inferred [49]. The behaviour of both *Partitiviridae* and *Totiviridae* (Fig. 1) closely resembles the cospeciation-duplication pattern depicted on p. 44 in [75], but with a higher ratio of codivergences to duplications. This is in agreement with the observed host distribution and the individual ParaFit tests (Figures 4, 5), as codivergence appears dominant, but some host taxa have been colonized at least twice independently. Accordingly, the topology of larger subtrees is always largely, but seldom entirely identical to the topology of the corresponding host trees. While Fahrenholz' rule of strict codivergence [37] can thus be rejected, both *Partitiviridae* and *Totiviridae* correspond to a pattern called ‘deep co-phylogeny’ [50], i.e. the presence of large parasite subtrees which potentially include few insignificant links to other host groups but whose majority of members is characterized by significant links to hosts exclusively belonging a certain host clade. These host clades are *Ascomycota* (*Pezizomycotina*), *Basidiomycota* and *Viridiplantae* for *Partitiviridae* (Fig. 4) and *Ascomycota*, *Euglenozoa*, *Metazoa* and *Viridiplantae* for *Totiviridae* (Fig. 5).

Our results for these two families also shed light on the question whether some viruses described as plant pathogens are actually mycoviruses of the plant host's fungal endophytes, as suggested by [23]. Codivergence between plants and the mycoviruses of their endophytic fungi would require codivergence between the plant and the endophyte on the one hand and codivergence between the endophyte and mycoviruses on the other. While such a scenario is not impossible, particularly considering the absence of natural vectors of mycoviruses [4], [5], which might decrease resource tracking [44], it is clearly less parsimonious than the assumption that these viruses parasite the plants themselves. The congruent subtrees of plants and viruses, particularly in the case of *Partitiviridae*, thus provide some counter-evidence for the mycovirus/endophyte hypothesis for these viruses. On the other hand, observing single plant hosts within subtrees comprising mycoviruses (e.g., ‘Grapevine-associated Partitivirus 2’ in Fig. 4), might indicate in some cases that the host specificity of these viruses should be reassessed.

In contrast, exploring the cost space for *Narnaviridae* resulted in a pattern which is most similar, but not identical, to the duplication-switching pattern shown on p. 45 in [75]. The main difference is that p values not larger than α = 0.05 are also observed for high switching costs (Fig. 1), i.e. the dominant event in *Narnaviridae* evolution is duplication, not switching. This is in agreement with the high number of viruses sampled from the same hosts, particularly *Cryphonectria cubensis* (Fig. 3). However, such host distributions are likely to mainly reflect the research interest in this plant-pathogenic fungus [13], [14], [20] and not the real host distribution of *Narnaviridae*. Because of the significant correlation between total sampling size for each virus family and the respective proportion of significant associations detected, above we already warned against the over-interpretation of the outcomes of the co-phylogenetic tests for the families *Chrysoviridae*, *Endornaviridae*, *Hypoviridae* and *Narnaviridae*.

While the currently small sample size even prevented TreeFitter analysis of *Endornaviridae* and *Hypoviridae*, it most likely also caused the difficulties in interpreting the TreeFitter cost space exploration result for *Chrysoviridae*. In fact, none of the tested parameter combinations yielded the rejection of the null hypothesis that the reconstruction of host-parasite evolution reconstruction is not better (i.e., more parsimonious) than random. Moreover, the resulting pattern can not be assigned to any of Ronquist's schemes [75]. While the two plant hosts included in this dataset might be due to wrongly annotated hosts harbouring endophytic fungi with mycoviruses (see above), the remaining associations were not significant either (supplementary file S2). Some congruent subtrees are apparent in Fig. 2 such as, e.g. the sister-group relationship of the viruses from *Neosartorya fumigata* and *Penicillium chrysogenum*, and among the parasites annotated as mycoviruses, only the position of ‘*Fusarium oxysporum* chrysovirus 1’ is apparently not in accordance with the one of its host. In the case of such small datasets, annotation errors regarding the association can easily lead to accepted global null hypotheses of random host-parasite relationships, a problem that does not occur in large-scale co-phylogenetic analyses [48], [50], [51].

We thus hypothesize that all families comprising mycoviruses evolve in basically the same manner relative to their hosts and that the observed differences between the examined virus families are caused by insufficient sampling for all of them except the two largest ones. Furthermore, while mainly significant associations were observed between fungal hosts and the better sampled families *Partitiviridae* and *Totiviridae*, this also holds for the non-fungal hosts in these two datasets. That is, there is currently no reason to assume that the host-parasite evolution of mycoviruses follows other principles than the one of the parasites of non-fungal hosts nested within the same families. Hence, one might interpret the congruence between mycovirus and host phylogenies as indicative of a little probability of lateral transfer between hosts, based on the assumption that otherwise resource tracking would be dominant and decrease the agreement between the trees [44]. However, one would then have to accept that closely related viruses on other hosts are not different in this respect. Apparently, the lack of (known) natural vectors alone [3], [5] is insufficient to cause higher levels of codivergence in mycoviruses than in other viruses.

### Conclusion

To the best of our knowledge, in this study we have presented the first analysis of codivergence between mycoviruses and their hosts, using state-of-the-art statistical co-phylogenetic tests and assessing the sensitivity of the results to the settings used for the inference of host and parasite trees. The main co-phylogenetic patterns observed were quite stable, indicating that phylogenomics of viruses is feasible within families, but it might frequently be necessary to remove rogue taxa to improve resolution. Largely congruent mycovirus and host phylogenies were observed in only two of the examined viral families, but the remaining ones may simply be too sparsely sampled to allow the co-phylogenetic tests to detect topological congruence. Codivergence might be the dominant mode of divergence of both mycoviruses and their close relatives on other hosts, and ‘deep cophylogeny’ [50] might be the dominant distributional pattern of mycoviruses on their hosts, but we conclude that increased sampling of mycoviruses, particularly on as yet unconsidered fungi (e.g., those forming mycorrhiza [17], [89], [90]), is a prerequisite for a more in-depth assessment of this question. Improved taxon coverage might also shed more light on the evolutionary role of genes of mycoviral origin integrated in the host genome such as the totivirus-like sequences detected in the genomes of budding yeasts [91]. As in some plant-virus systems, the corresponding proteins might even increase the host's resistance to other viruses [92]. Because lateral transfer should be even less probably for such nuclear genomic copies of viruses, according to Timm's [44] rule co-phylogenetic congruence is expected to increase in subgroups of viruses that underwent integration into the host genome. State-of-the-art sequencing technology used in screening for viral sequences is likely to greatly improve our knowledge on the diversity and host distribution of these viruses [23], but cultivating the hosts might frequently be necessary to elucidate difficult systems such as those involving fungal endophytes.

## Supporting Information

File S1 Details on the functions used for inferring distances from the host classification.
(PDF)

File S2 Contains (i) the list of the Genbank accessions for all examined viruses; (ii) general features of the obtained supermatrices (total numbers of characters, viruses, corresponding numbers of hosts, numbers and annotations of clusters of orthologs present in each supermatrix) and of the resulting trees (log likelihood of the best tree found, and bootstrap support averaged over all branches) for all examined virus families and method settings; (iii) the ParaFit test results for all individual host-virus associations under all conditions; (iv) a summary of the ParaFit test results referred to in the text.
(XLS)

File S3 Presence and absence of genes in viral genomes and in clusters of orthologs for all virus families visualized as heatmaps. The reasons for the exclusion of each virus genome and cluster of orthologs that was deleted prior to phylogenetic analysis are indicated.
(PDF)
