# Supplementary material for: Codivergence of Mycoviruses with Their Hosts
Source: PLoS One. 2011 Jul 29;6(7):e22252. doi: 10.1371/journal.pone.0022252 (PMC3146478; doi:10.1371/journal.pone.0022252)
Supplement: File S1 — Details on the functions used for inferring distances from the host classification. (PDF) [file pone.0022252.s001.pdf]

“Codivergence of mycoviruses with their hosts”
Supplementary file 1:
(Non-)Ultrametricity of taxonomic distance functions

0. Introduction

A distance function d is said to be ultrametric if the equation

$$d(X,Y) \leq max(d(X,Z), d(Y,Z)) \quad \text{(S1.0.1)}$$

holds for any three items X, Y and Z (Johnson 1967) between which a distance d is defined. In other words, the two larger distances from all distance triplets are identical. The study “Codivergence of mycoviruses with their hosts” defines four distance functions derived from a classification, patristic ones (d_P), 'quasi-patristic' distances (d_Q), 'theory' distances (d_T), and 'first mismatch' distances (d_F). Here we show that the former two functions usually do not yield ultrametric distances but the latter two do.

1. Patristic and quasi-patristic distances are not necessarily ultrametric, but quasi-patristic distances less deviate from ultrametricity

To measure the deviation from ultrametricity, we devised a triplet-based statistics, inspired by the quartet-based measurement of deviations from additivity ('δ-values') as used in Auch et al. (2006). For each triplet of items X, Y and Z we define

$$u(X,Y,Z) := max(d(X,Y), d(X,Z), d(Y,Z)) - median(d(X,Y), d(X,Z), d(Y,Z)) \quad \text{(S1.1.1)},$$

i.e. the difference between the largest and the 2nd largest distance value. If the distance matrix is ultrametric, $u(X, Y, Z)$ is zero for all triplets X, Y and Z. If all items T_n are indexed by $1 \leq n \leq N$, the number of non-ordered triplets is $M := N \times (N-1) \times (N-2)/6$, and the rescaled average deviation from ultrametricity can be defined as

$$u_{ra} := \left(\sum_{i<j<k} u(T_i, T_j, T_k)\right) / (M \cdot max(d(T_i, T_j), i<j)) \quad \text{(S1.1.2)}.$$

Calculating the average allows one to compare matrices comprising distinct numbers of items, whereas scaling by division through the maximal distance allows the comparison of matrices of distances on a distinct original scale. In effect, the distances are compared as if subjected to ranging beforehand (Legendre and Legendre 1998, p. 38), hence $0 \leq u_{ra} < 1$ for any distance matrix.

The following Table S1.1 contains the u_{ra} values for the five virus families (using the largest host distance matrix for each family only to avoid redundancy) and the four distance formulas examined. The calculations were performed using the diststats program (Auch et al. 2006), to which code for the inference of triplet statistics was added. It was also used to calculate δ-values (Auch et al. 2006). Bound between 0 and 1, lower δ-values indicate higher treelikeness; for additive distances, $\delta = 0$ holds.

Family	Distance formula	δ-value	u_{ra}
Chrysoviridae	first mismatch	0.00E+000	0.00E+000
	patristic	0.00E+000	4.17E-002

	quasi-patristic	1.13E-001	1.16E-002
	theory	0.00E+000	0.00E+000
Endornaviridae	first mismatch	0.00E+000	0.00E+000
	patristic	0.00E+000	6.93E-002
	quasi-patristic	4.27E-002	1.72E-002
	theory	0.00E+000	0.00E+000
Narnaviridae	first mismatch	0.00E+000	0.00E+000
	patristic	0.00E+000	4.87E-002
	quasi-patristic	8.45E-002	2.40E-002
	theory	0.00E+000	0.00E+000
Partitiviridae	first mismatch	0.00E+000	0.00E+000
	patristic	0.00E+000	4.54E-002
	quasi-patristic	4.08E-002	1.14E-002
	theory	0.00E+000	0.00E+000
Totiviridae	first mismatch	0.00E+000	0.00E+000
	patristic	0.00E+000	6.02E-002
	quasi-patristic	9.81E-002	2.30E-002
	theory	0.00E+000	0.00E+000

Apparently, the deviation from ultrametricity of 'quasi-patristic' distances is usually at most half as high as the one of patristic distances. While we do not offer a mathematical proof here, we opine that this result can be generalized because the only possible reason for the non-ultrametricity of distances derived from taxonomic classifications is the presence of distinct numbers of parent taxa, and 'quasi-patristic' distances are specifically rescaled to correct for this effect.

2. 'Theory' distances are ultrametric if the dataset is restricted to the tips

As shown in Table S1.1, 'theory' distances yielded strictly ultrametric distances for the dataset under study. We now demonstrate that this holds for terminal taxa in all kinds of taxonomic classifications. Under the conditions that the classification is restricted to the terminal taxa of interest and their parent taxa, and that we calculate d_T only between terminal taxa, the denominator L in formula (3) of the main text becomes constant:

$$L := \log(p(X)) + \log(p(Y)) = \log(1/N) + \log(1/N) = -2 \cdot \log(N) \quad \text{(S1.2.1)},$$

where N is the number of terminal taxa. The relevant equation (combining S1.0.1 and formula 3 from the main text) then simplifies to

$$1 - 2 \cdot \log(p(s(X,Y)))/L \leq max(1 - 2 \cdot \log(p(s(X,Z)))/L, 1 - 2 \cdot \log(p(s(Y,Z)))/L) \quad \text{(S1.2.2)},$$

which yields

$$p(s(X,Y)) \geq min(p(s(X,Z)), p(s(Y,Z))) \quad \text{(S1.2.3)}.$$

Here, it is crucial to note that two taxa A and B are more closely related to each other than to a third taxon C if and only if there is at least one parent taxon of both A and B which is not a parent taxon of C.

If so, *s(A,B)* is such as parent taxon (as the *smallest* taxon including both A and B, it is even the parent taxon most likely to not include *C*). Moreover, because in a hierarchical classification such as the Linnean system each taxon can possess at most a single immediate parent taxon, it follows that

$$s(A,C)=s(s(A,B),C)=s(B,C) \quad \text{(S1.2.4)}$$

and

$$p(s(A,C))=p(s(B,C))\geq p(s(A,B)) \quad \text{(S1.2.5)}$$

thus proofing S1.2.3.

3. 'First mismatch' distances are ultrametric

Table S1.1 shows that 'first mismatch' distances yielded strictly ultrametric distances for the datasets under study. This result can be generalized as follows. The relevant equation (combining S1.0.1 and formula 4 from the main text) is

$$1/(1+|t(X)\cap t(Y)|)\leq max(1/(1+|t(X)\cap t(Z)|), 1/(1+|t(Y)\cap t(Z)|)) \quad \text{(S1.3.1).}$$

It follows from S1.3.1 that

$$|t(X)\cap t(Y)|\geq min(|t(X)\cap t(Z)|, |t(Y)\cap t(Z)|) \quad \text{(S1.3.2).}$$

Again, it is important to note that two taxa *A* and *B* are more closely related to each other than to a third taxon *C* if and only if there is at least one parent taxon *P* of *A* and *B* which is not a parent taxon of *C*. In other words, *A* and *B* have more parent taxa in common than either *A* and *C*, or *B* and *C*. Moreover, it follows that

$$t(A)\cap t(C)=t(P)\cap t(C)=t(B)\cap t(C) \quad \text{(S1.3.3).}$$

Accordingly, if (i) *X*, *Y* and *Z* are equally closely related to each other then all three intersections have equal sizes; if (ii) *X* and *Y* are more closely related to each other than to *Z* then the intersection of *t(X)* and *t(Y)* is larger than both other intersections; (iii) if *X* is more closely related to *Z* than to *Y* then the intersection of *t(X)* and *t(Y)* has the same size than the intersection of *t(Z)* and *t(Y)* and the intersection of *t(X)* and *t(Z)* is larger; and (iv) if *Y* is more closely related to *Z* than to *X* then the intersection of *t(X)* and *t(Y)* has the same size than the intersection of *t(X)* and *t(Z)* and the intersection of *t(Y)* and *t(Z)* is larger.

References

All references were also cited in the main manuscript.
