# Supplementary material for: Codivergence of Mycoviruses with Their Hosts
Source: PLoS One. 2011 Jul 29;6(7):e22252. doi: 10.1371/journal.pone.0022252 (PMC3146478; doi:10.1371/journal.pone.0022252)
Supplement: File S3 — Presence and absence of genes in viral genomes and in clusters of orthologs for all virus families visualized as heatmaps. The reasons for the exclusion of each virus genome and cluster of orthologs that was deleted prior to phylogenetic analysis are indicated. (PDF) [file pone.0022252.s003.pdf]

“Codivergence of mycoviruses with their hosts”
Supplementary file 3:
Gene content visualized as heatmaps

In order to visualize the distribution of genes over the viral genomes and clusters of orthologs we calculated heatmaps using the eponymous function in R (R 2002). Input matrices contained the viruses as rows and the clusters of orthologs as columns. The fields contained 0 to indicate absence, 1 to indicate presence, respectively. The formula suggested by Russell and Rao (1940; see Legendre and Legendre 1998, p. 257) was used to as distance function between rows and columns, respectively; this formula is equivalent to the number of double presences divided by the total number of fields. The inverse of the number of positive entries per row or column was used to reorder the dendrograms after clustering (for details, see the documentation of *heatmap()* in R). The matrices were drawn without re-scaling, using light yellow for presence, red for absence. The clusters of orthologs that had to be removed because they contained genes from less than four distinct viruses, as well as the viruses that had to removed because they had no genes co-occurring in clusters of orthologs with the majority of the viruses were marked with an asterisk. For each virus family, one plot was generated.

References

Legendre P, Legendre L (1998) Numerical ecology. 2nd ed. Amsterdam, Elsevier.

R (2002) The R Project for Statistical Computing. Available online at http: //www.r-project.org/ .

Chysoviridae

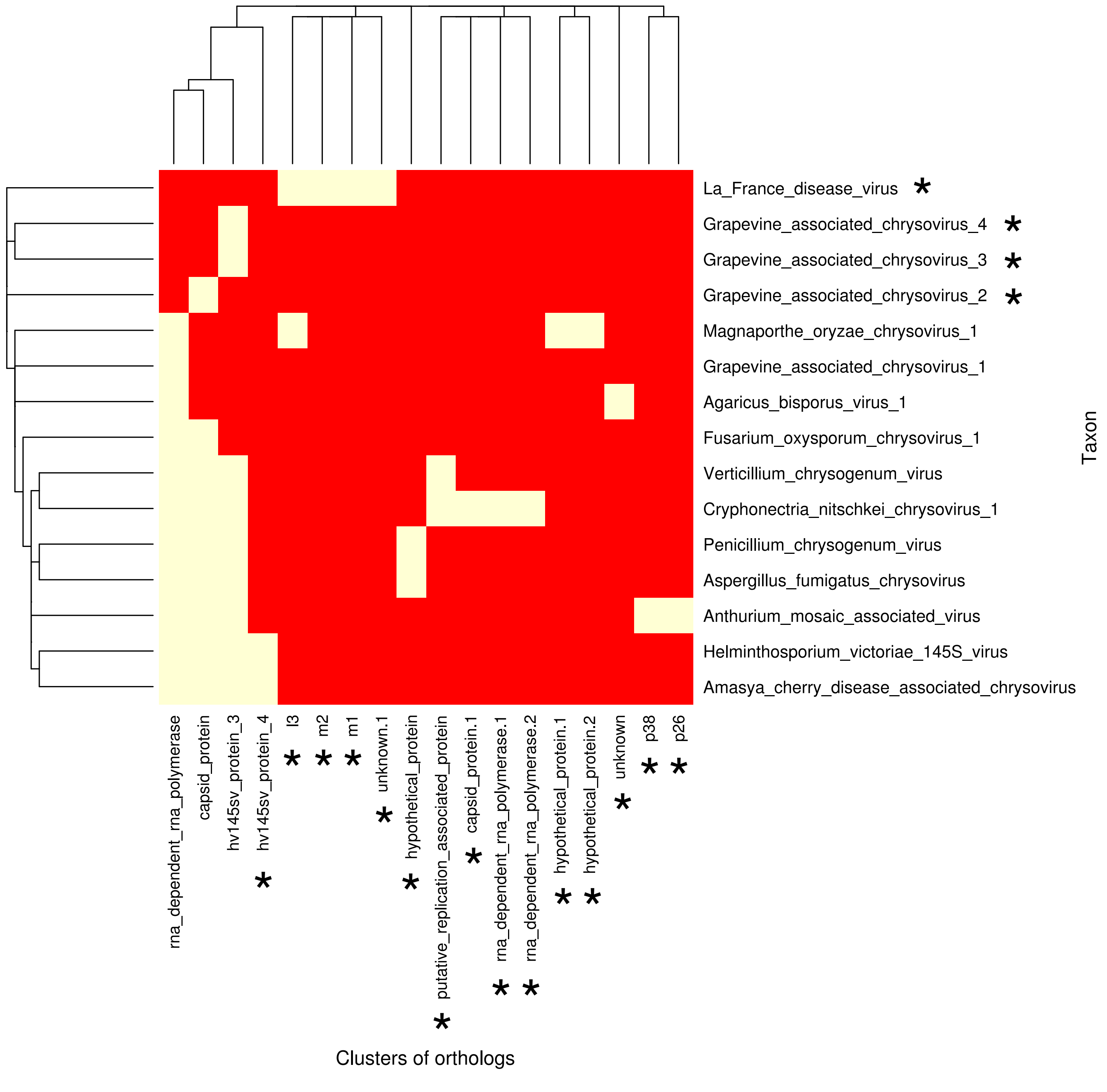

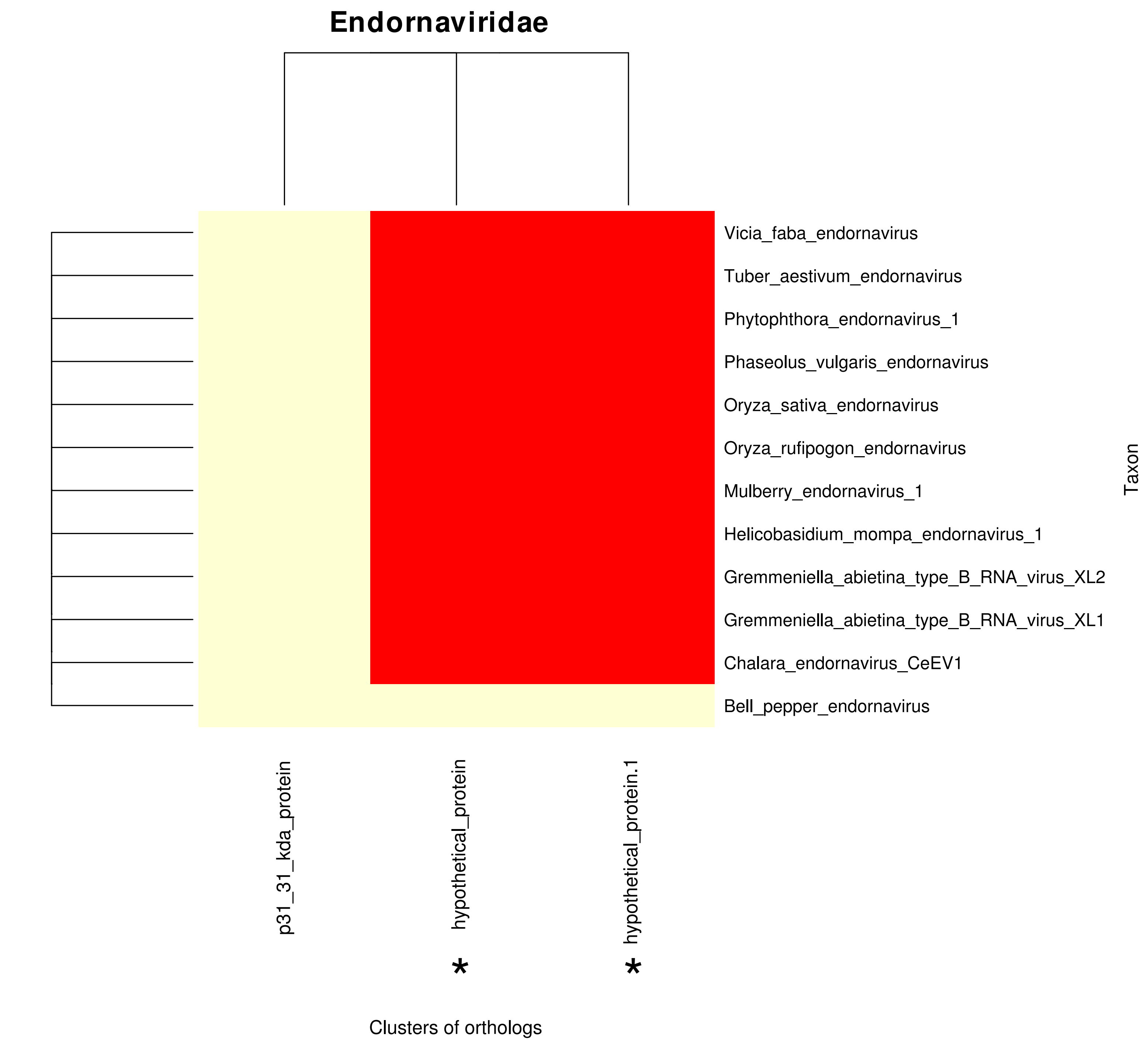
Endornaviridae
Vicia_faba_endornavirus
Tuber_aestivum_endornavirus
Phytophthora_endornavirus_1
Phaseolus_vulgaris_endornavirus
Oryza_sativa_endornavirus
Oryza_rufipogon_endornavirus
Mulberry_endornavirus_1
Helicobasidium_mompa_endornavirus_1
Gremmeniella_abietina_type_B_RNA_virus_XL2
Gremmeniella_abietina_type_B_RNA_virus_XL1
Chalara_endornavirus_CeEV1
Bell_pepper_endornavirus
Taxon
p31_31_kda_protein
hypothetical_protein
hypothetical_protein.1
*
*
Clusters of orthologs

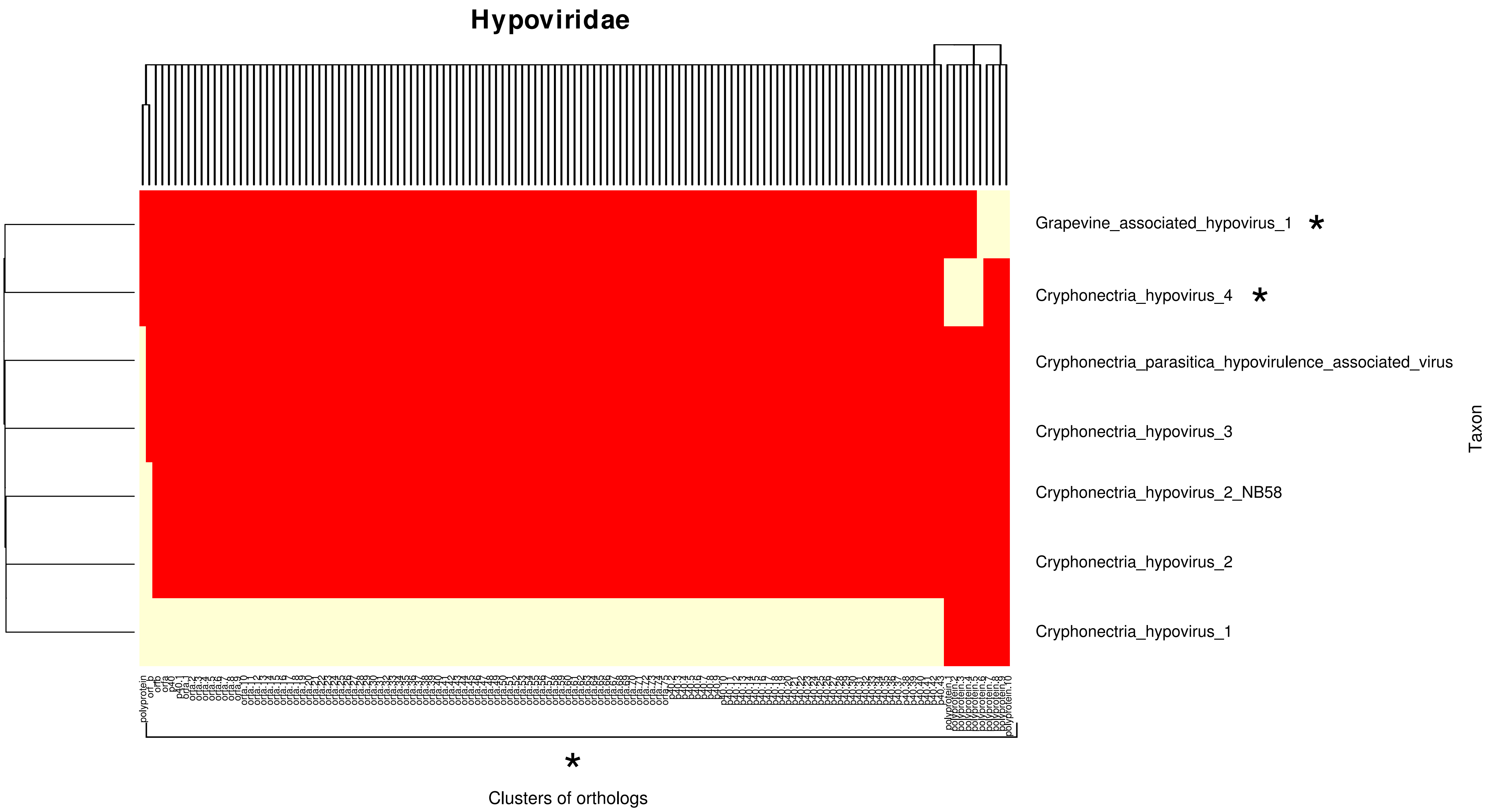
Hypoviridae
Grapevine_associated_hypovirus_1 *
Cryphonectria_hypovirus_4 *
Cryphonectria_parasitica_hypovirulence_associated_virus
Cryphonectria_hypovirus_3
Cryphonectria_hypovirus_2_NB58
Cryphonectria_hypovirus_2
Cryphonectria_hypovirus_1
Taxon
*
Clusters of orthologs

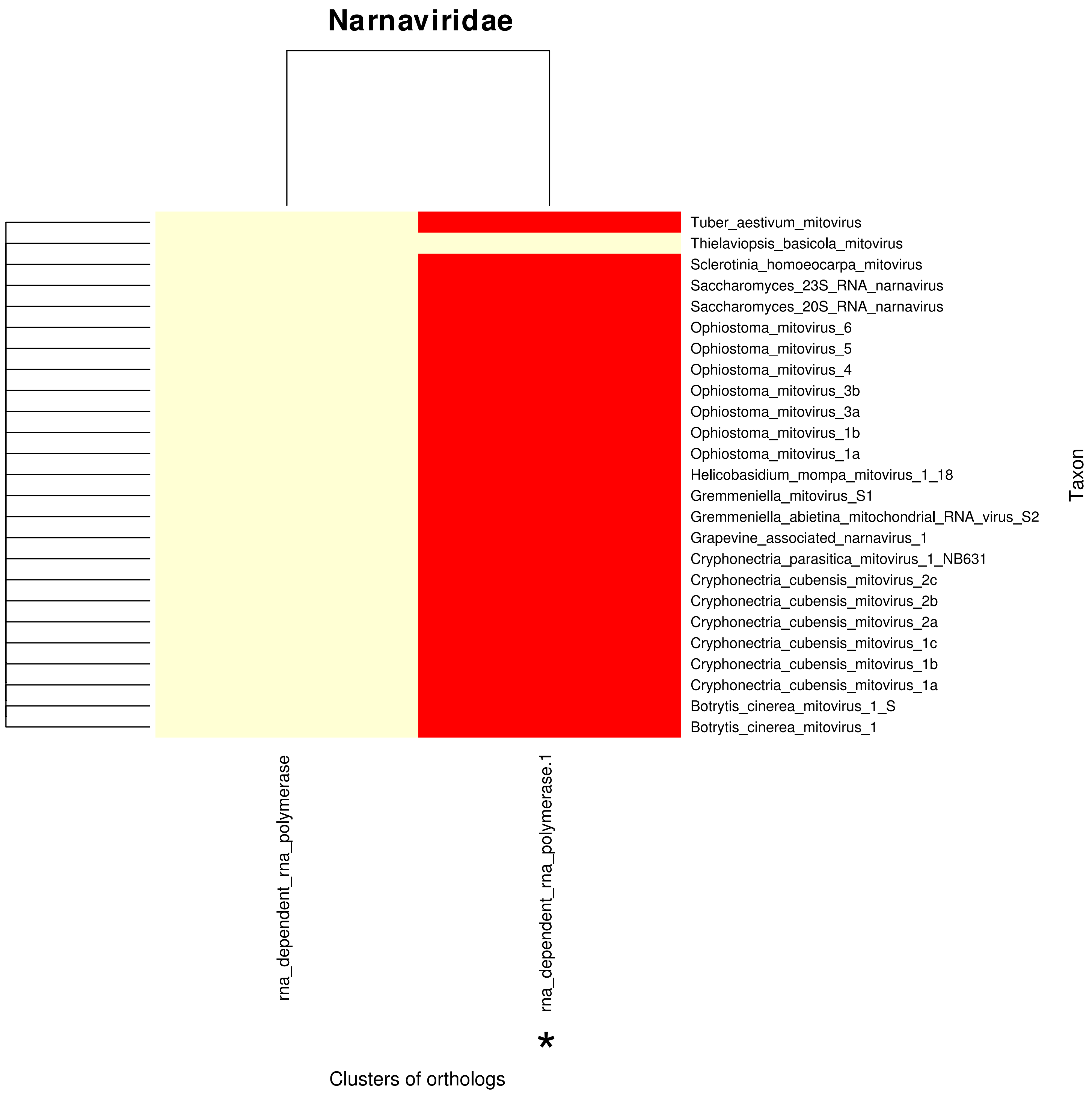
Narnaviridae
Tuber_aestivum_mitovirus
Thielaviopsis_basicola_mitovirus
Sclerotinia_homoeocarpa_mitovirus
Saccharomyces_23S_RNA_narnavirus
Saccharomyces_20S_RNA_narnavirus
Ophiostoma_mitovirus_6
Ophiostoma_mitovirus_5
Ophiostoma_mitovirus_4
Ophiostoma_mitovirus_3b
Ophiostoma_mitovirus_3a
Ophiostoma_mitovirus_1b
Ophiostoma_mitovirus_1a
Helicobasidium_mompa_mitovirus_1_18
Gremmeniella_mitovirus_S1
Gremmeniella_abietina_mitochondrial_RNA_virus_S2
Grapevine_associated_narnavirus_1
Cryphonectria_parasitica_mitovirus_1_NB631
Cryphonectria_cubensis_mitovirus_2c
Cryphonectria_cubensis_mitovirus_2b
Cryphonectria_cubensis_mitovirus_2a
Cryphonectria_cubensis_mitovirus_1c
Cryphonectria_cubensis_mitovirus_1b
Cryphonectria_cubensis_mitovirus_1a
Botrytis_cinerea_mitovirus_1_S
Botrytis_cinerea_mitovirus_1
Taxon
rna_dependent_rna_polymerase
rna_dependent_rna_polymerase.1
*
Clusters of orthologs

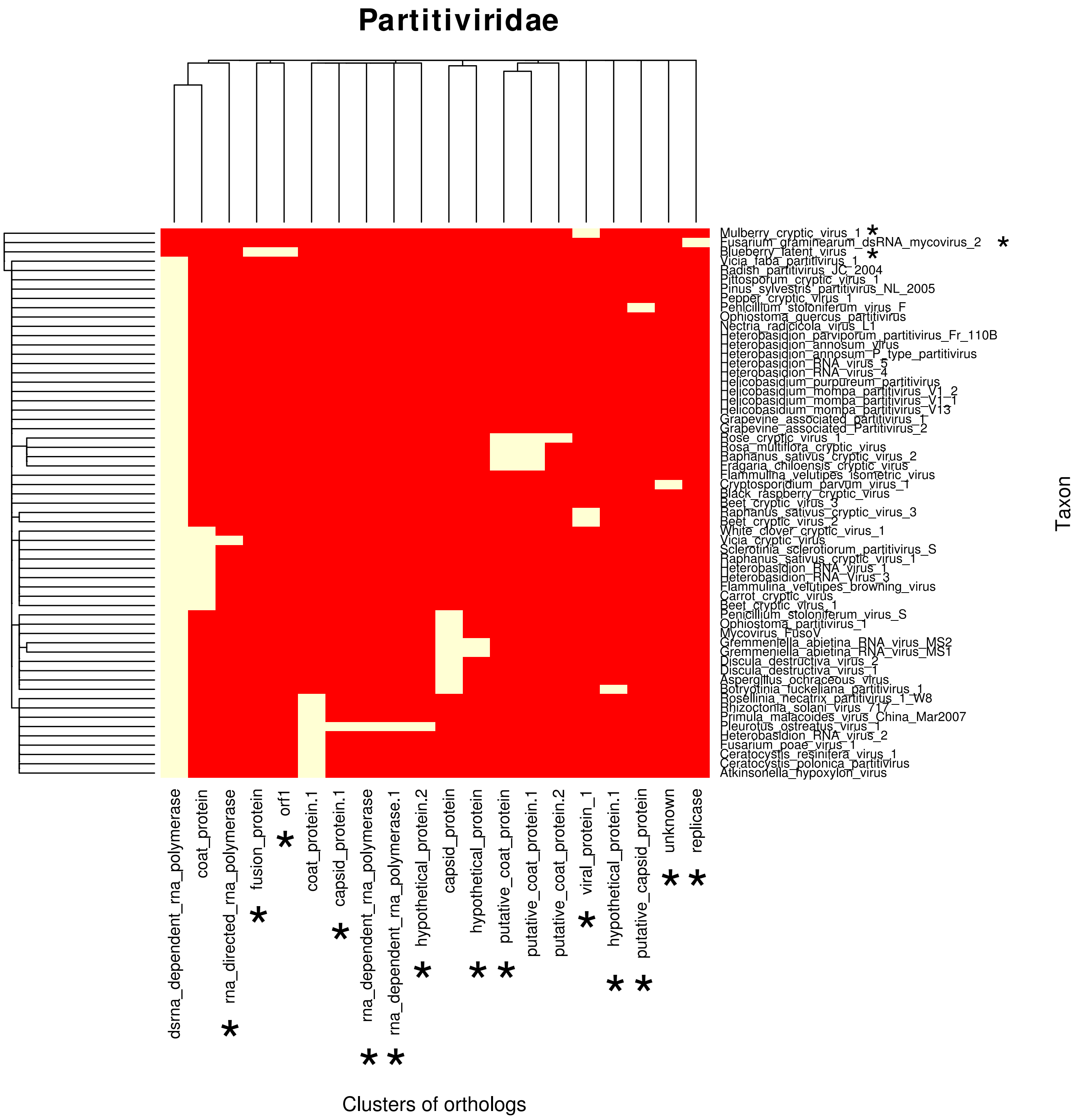
Partitiviridae
Mulberry_cryptic_virus_1
Fusarium_graminearum_dsRNA_mycovirus_2
Blueberry_latent_virus
Vicia_faba_partitivirus_1
Radish_partitivirus_JC_2004
Pittosporum_cryptic_virus_1
Pinus_sylvestris_partitivirus_NL_2005
Pepper_cryptic_virus_1
Penicillium_stoloniferum_virus_F
Ophiostoma_quercus_partitivirus
Nectria_radicicola_virus_L1
Heterobasidion_parviporum_partitivirus_Fr_110B
Heterobasidion_annosum_virus
Heterobasidion_annosum_P_type_partitivirus
Heterobasidion_RNA_virus_5
Heterobasidion_RNA_virus_4
Helicobasidium_purpureum_partitivirus
Helicobasidium_mompa_partitivirus_V1_2
Helicobasidium_mompa_partitivirus_V1_1
Helicobasidium_mompa_partitivirus_V13
Grapevine_associated_partitivirus_1
Grapevine_associated_Partitivirus_2
Rose_cryptic_virus_1
Rosa_multiflora_cryptic_virus
Raphanus_sativus_cryptic_virus_2
Fragaria_chiloensis_cryptic_virus
Flammulina_velutipes_isometric_virus
Cryptosporidium_parvum_virus_1
Black_raspberry_cryptic_virus
Beet_cryptic_virus_3
Raphanus_sativus_cryptic_virus_3
Beet_cryptic_virus_2
White_clover_cryptic_virus_1
Vicia_cryptic_virus
Sclerotinia_sclerotiorum_partitivirus_S
Raphanus_sativus_cryptic_virus_1
Heterobasidion_RNA_virus_1
Heterobasidion_RNA_virus_3
Flammulina_velutipes_browning_virus
Carrot_cryptic_virus
Beet_cryptic_virus_1
Penicillium_stoloniferum_virus_S
Ophiostoma_partitivirus_1
Mycovirus_FusoV
Gremmeniella_abietina_RNA_virus_MS2
Gremmeniella_abietina_RNA_virus_MS1
Discula_destructiva_virus_2
Discula_destructiva_virus_1
Aspergillus_ochraceous_virus
Botryotinia_fuckeliana_partitivirus_1
Rosellinia_necatrix_partitivirus_1_W8
Rhizoctonia_solani_virus_717
Primula_malacoides_virus_China_Mar2007
Pleurotus_ostreatus_virus_1
Heterobasidion_RNA_virus_2
Fusarium_poae_virus_1
Ceratocystis_resinifera_virus_1
Ceratocystis_polonica_partitivirus
Atkinsonella_hypoxylon_virus
Taxon
dsrna_dependent_rna_polymerase
coat_protein
rna_directed_rna_polymerase
fusion_protein
orf1
coat_protein.1
capsid_protein.1
rna_dependent_rna_polymerase
rna_dependent_rna_polymerase.1
hypothetical_protein.2
capsid_protein
hypothetical_protein
putative_coat_protein
putative_coat_protein.1
putative_coat_protein.2
viral_protein_1
hypothetical_protein.1
putative_capsid_protein
unknown
replicase
Clusters of orthologs

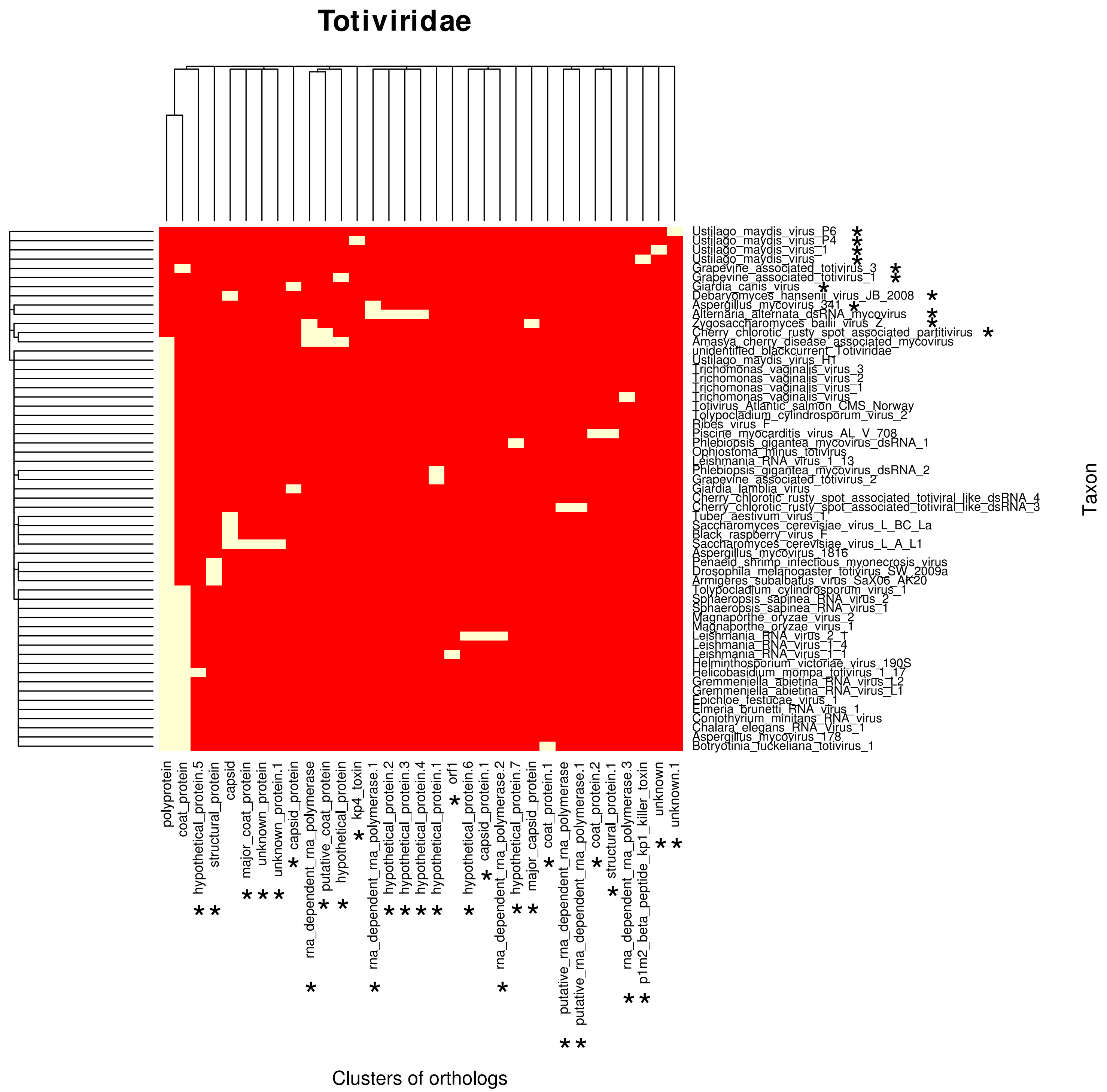
Totiviridae
Ustilago_maydis_virus_P6
Ustilago_maydis_virus_P4
Ustilago_maydis_virus_1
Ustilago_maydis_virus
Grapevine_associated_totivirus_3
Grapevine_associated_totivirus_1
Giardia_canis_virus
Debaryomyces_hansenii_virus_JB_2008
Aspergillus_mycovirus_341
Alternaria_alternata_dsRNA_mycovirus
Zygosaccharomyces_bailii_virus_Z
Cherry_chlorotic_rusty_spot_associated_partitivirus
Amasya_cherry_disease_associated_mycovirus
unidentified_blackcurrent_Totiviridae
Ustilago_maydis_virus_H1
Trichomonas_vaginalis_virus_3
Trichomonas_vaginalis_virus_2
Trichomonas_vaginalis_virus_1
Trichomonas_vaginalis_virus
Totivirus_Atlantic_salmon_CMS_Norway
Tolypocladium_cylindrosporum_virus_2
Ribes_virus_F
Piscine_myocarditis_virus_AL_V_708
Phlebiopsis_gigantea_mycovirus_dsRNA_1
Ophiostoma_minus_totivirus
Leishmania_RNA_virus_1_13
Phlebiopsis_gigantea_mycovirus_dsRNA_2
Grapevine_associated_totivirus_2
Giardia_lamblia_virus
Cherry_chlorotic_rusty_spot_associated_totiviral_like_dsRNA_4
Cherry_chlorotic_rusty_spot_associated_totiviral_like_dsRNA_3
Tuber_aestivum_virus_1
Saccharomyces_cerevisiae_virus_L_BC_La
Black_raspberry_virus_F
Saccharomyces_cerevisiae_virus_L_A_L1
Aspergillus_mycovirus_1816
Penaeid_shrimp_infectious_myonecrosis_virus
Drosophila_melanogaster_totivirus_SW_2009a
Armigeres_subalbatus_virus_SaX06_AK20
Tolypocladium_cylindrosporum_virus_1
Sphaeropsis_sapinea_RNA_virus_2
Sphaeropsis_sapinea_RNA_virus_1
Magnaporthe_oryzae_virus_2
Magnaporthe_oryzae_virus_1
Leishmania_RNA_virus_2_1
Leishmania_RNA_virus_1_4
Leishmania_RNA_virus_1_1
Helminthosporium_victoriae_virus_190S
Helicobasidium_mompa_totivirus_1_17
Gremmeniella_abietina_RNA_virus_L2
Gremmeniella_abietina_RNA_virus_L1
Epichloe_festucae_virus_1
Eimeria_brunetti_RNA_virus_1
Coniothyrium_minitans_RNA_virus
Chalara_elegans_RNA_Virus_1
Aspergillus_mycovirus_178
Botryotinia_fuckeliana_totivirus_1
Taxon
polyprotein
coat_protein
hypothetical_protein.5
structural_protein
capsid
major_coat_protein
unknown_protein
unknown_protein.1
capsid_protein
rna_dependent_rna_polymerase
putative_coat_protein
hypothetical_protein
kp4_toxin
rna_dependent_rna_polymerase.1
hypothetical_protein.2
hypothetical_protein.3
hypothetical_protein.4
hypothetical_protein.1
orf1
hypothetical_protein.6
capsid_protein.1
rna_dependent_rna_polymerase.2
hypothetical_protein.7
major_capsid_protein
coat_protein.1
putative_rna_dependent_rna_polymerase
putative_rna_dependent_rna_polymerase.1
coat_protein.2
structural_protein.1
rna_dependent_rna_polymerase.3
p1m2_beta_peptide_kp1_killer_toxin
unknown
unknown.1
Clusters of orthologs
